# Synthesis and Characterization of New Composite Materials Based on Magnesium Phosphate Cement for Fluoride Retention

**DOI:** 10.3390/ma16020718

**Published:** 2023-01-11

**Authors:** Sana Gharsallah, Abdulrahman Alsawi, Bechir Hammami, Mohamed Khitouni, Clarence Charnay, Mahmoud Chemingui

**Affiliations:** 1 Laboratory of Inorganic Chemistry, LR17-ES-07, Faculty of Science, University of Sfax, Sfax 3018, Tunisia; 2 Department of Physics, College of Science, Qassim University, Buraidah 51452, Saudi Arabia; 3 Department of Chemistry, College of Science, Qassim University, Buraidah 51452, Saudi Arabia; 4 Charles Gerhard Institut, UMR-5253 CNRS-UM-ENSCM, University of Montpellier, Place E, Bataillon, CEDEX 5, F-34095 Montpellier, France

**Keywords:** magnesium phosphate cements (MPC), alumina, fluoride, adsorption, isotherms

## Abstract

In this research work, new composite materials based on magnesium phosphate cement (MPC) were developed to evaluate the retention of fluorine from wastewater. This material was prepared with dead burned magnesia oxide (MgO), ammonium dihydrogen phosphate (NH_4_H_2_PO_4_), and some retarding agents. We chose to synthesize with hydrogen peroxide instead of water; alumina and zeolite were also added to the cement. The obtained optimal conditions were studied and analyzed by X-ray diffraction (XRD) and scanning electron microscopy (SEM), Fourier transform infrared spectroscopy (FTIR), BET, and thermogravimetric analysis (TGA). The adsorbents showed a strong ability to remove fluoride from contaminated water, and the best defluoridation capacity was evaluated as 2.21 mg/g for the H_2_O_2_ cement. Equilibrium modeling was performed, and the experimental data were presented according to the isotherms of Langmuir, Freundlich, Temkin, and Dubinin–Radushkevich.

## 1. Introduction

Magnesium phosphate cements (MPC) are chemical cements derived from the family of phosphate-bound cements. Curing is the result of a chemical reaction between basic magnesia and an acid solution including phosphates to which various components may be added [1].

This cement is obtained from a mixture of magnesia MgO) and ammonium dihydrogen phosphate in the presence of water, expressed as

MgO + NH_4_H_2_PO_4_ + 5H_2_O → MgNH_4_ PO_4_.6H_2_O (struvite) (1)

In fact, the growth of struvite is a rapid process resulting from the reaction of magnesia and phosphate [2]. Throughout this reaction, salts are formed through heavy metals, which improve their chemical stability. In addition, the microscopic reaction grants the cement matrix a porous structure, which helps improve the adsorption and the encapsulation of heavy metals [1]. According to numerous studies, the properties of cement are largely affected by several parameters: the molar ratio of magnesium to phosphate (M/P), the reactivity of magnesia, the amount of added water [3], the use of setting retarders, and the initial concentration of phosphate. This type of cement has attracted the widest interest, referring to its outstanding properties such as fast setting time, adhesive properties, low permeability, accelerated resistance development, excellent bonding with almost any clean and dry surface, high strength at an early stage, fast hydration process, fire-resistance properties, lower drying shrinkage [4], great volume stability, strong bonding force, long durability, and high heat and temperature resistance, and the MPC presents a special environmentally friendly adaptability [5]. Relying upon these properties, cement displays a wide range of applications such as rapid-repair materials for deteriorated bridge decks, highways, and airport runways; the rehabilitation of damaged civil structures; biomedical materials design; bone restoration; the stabilization of toxic matters and nuclear wastes; and the solidification and stabilization of radioactive wastes and heavy metallic ions. The basic drawback of this type of cement resides in its low stability in water [6]. In this research work, a new type of binder is formed using hydrogen peroxide as a solvent. The synthesized material has better resistance with the presence of porosity. In addition, two composites are synthesized using this cementitious matrix by introducing zeolite and aluminum oxide. The binders formed are used for the retention of fluorine and to improve the resistance (it has better water resistance even after a few days). Zeolite is a macroporous aluminosilicate mineral with a cage-like structure channels and cavities. The mechanisms of fluoride retention using zeolite can be described by occlusion and ion exchange. In addition, fluoride has an ionic radius of approximately 1.34Å, which allows it to be easily occluded in the zeolite cavity [7]. Moreover, the presence of phosphate and magnesium ions demonstrates a positive influence in the retention of fluorine.

In different regions of the world, fluoride corresponds to considerable natural contaminants in potable water. Despite the fact that it is found in air, water, animals, and plants, fluoride is frequently added at a rate of approximately 1 mg/L to drinking water supplies to prevent tooth decay [8]. The use of fluorine is extremely varied and affects several areas such as agriculture (being used as a fertilizer) and industry (being used as a semiconductor, in alumina electrolysis, in the production of high-purity graphite, etc.). However, the intrinsic problem lies when the concentration of fluoride exceeds 1.5 mg/L [8]. When it does, it generates fluorosis, which mainly affects the teeth and bones. In addition, it entails damage to the liver and causes endocrine glands, thyroid, cancer, infertility, brain damage, and Alzheimer’s syndrome [9]. Various adapted techniques have been reported for the defluoridation of water, such as ion exchange, nanofiltration, adsorption, reverse osmosis [10], electrocoagulation, and membrane separation. The most widely used technique for this treatment is adsorption as it provides great flexibility in design as well as in scaling operations, and owing to its moderate cost [11]. The most frequently used adsorbents for the selective removal of fluoride from water are activated carbon, polymeric resins, metal oxides, activated alumina [12,13], chitosan beads, and natural and synthetic-based biomaterials.

In this work, we chose a cement matrix containing alumina and zeolite; these materials have fluorine affinity, facilitating their fixation and improving adsorption to a great extent. In this research, we have found that the use of peroxide instead of water and a small amount of alumina or zeolite produces interesting results, especially in terms of creating porosity and increasing the hardness of the material and its resistance in water even after a few days. These interesting composites were tested for fluoride retention.

## 2. Experimental Procedure

MPC pastes were prepared through mixing powders, including magnesia (magnesium oxide) (MgO > 99%—Merck KGaA Frankfurter Str.25, Darmstadt, Germany), ADP (NH_4_H_2_PO_4_ > 99%—Sigma-Aldrich (Darmstadt, Germany)); borax, which was used as a set retarder; H_2_O_2_; aluminum oxide (alumina); and zeolite (13X). These substances were all provided by Sigma Aldrich. Magnesia was calcined at 1500 °C for 6 h to reduce its reactivity. A different quantity of renfort powder was inserted into the mixture. Aggregates were not used in this research, to prevent additional disruption from their impurities. The synthesis protocol consists of mixing magnesium and acid with the retarding agent, which is borax. We selected the suitable ratio which is Mg/P = 1 with merely sufficient amounts of solvent. For the pure cement, we used water as a solvent, while for the other samples 2 mL of H_2_O_2_ was used. Then, alumina or zeolite powders were added in an adequate and equal amount of 0.54 g, selected after a number of tests, and combined until a homogeneous paste was produced. The chemical compositions of synthesized samples are given in Table 1.

The crystalline phases of MPC pastes were identified using X-ray powder diffractometer X’per PRO PANalatycal (Philips, Farnborough, UK) with CuK_α_ radiation (λK_α_ = 1.54 Å), and the diffraction patterns were collected 10° < 2θ < 80°. The microstructure of the cement was characterized through scanning electron microscopy (SEM (JEOL)-Japon) equipped with an energy dispersive spectrometer (EDS). Brunauer, Emmett, and Teller (BET) (TriStar 3000 V6.06 A, UK) were used to determine the specific surface area and to examine the dehydration characteristics of struvite. Thermogravimetric analyses (TGA, Perkin-Elmer, Waltham, MA, USA) were conducted to determine the change in the mass of samples under sweeping air and a temperature rise ranging from 20 to 1150 °C, at heating rates of 20 °C/min. The functional groups and the bonding patterns in the prepared cement were characterized using Fourier transform infrared spectrophotometer (FTIR, Perkin-Elmer, USA) in the range of 450–4000 cm^−1^.

## 3. Results and Discussion

### 3.1. Pure and H_2_O_2_ Cements

**Figure 1:**
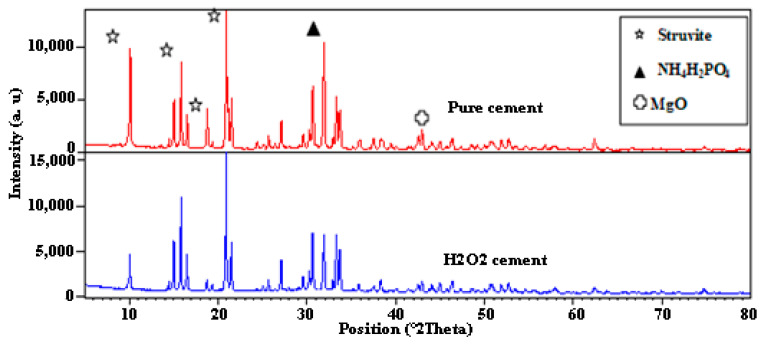
X-ray diffraction pattern of pure and H_2_O_2_ cements.

The X-ray diffraction patterns of pure and H_2_O_2_ cements are displayed in Figure 1. In the region of 10 to 21° -2-theta, a significant peak that is typical of struvite, the stable phase of cement can be seen, indicating the existence of crystallization. In order to maintain structural stability, the material uses peroxide, which benefits the cement by making it stronger and more water resistant. Along with the presence of peaks associated with residual NH_4_H_2_PO_4_ in the range of 30–33° 2-theta, small peaks related to unreacted magnesia were also noted in the neighborhood of 43° [4,6]. However, no new phase developed on the other side.

**Figure 2:**
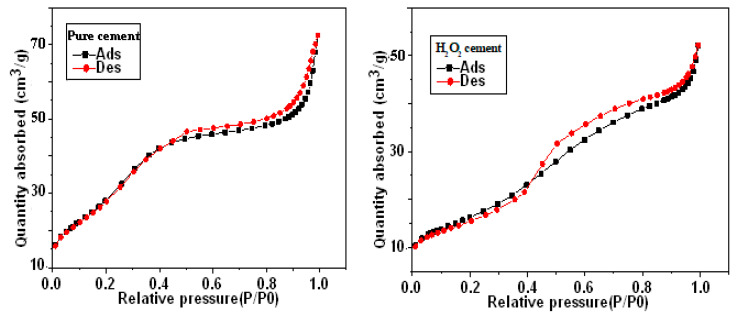
N2 adsorption/desorption curve for pure and H_2_O_2_ cements.

The N2 adsorption/desorption curve for pure and H_2_O_2_ cements is shown in Figure 2, and it produces the type II hysteresis curve that is most frequently observed. This graph demonstrates that multilayer adsorption and adsorption on non-porous surfaces start at high pressures. Furthermore, compared to a sample made up of split pores with a size distribution that is predominately in the microporous area, the hysteresis loop is of type H4. As seen in Figure 2, Brunauer, Emmett, and Teller (BET) reported a correct assessment of a particular surface. Pure cement has a BET surface of around 114.0695 m^2^/g, a pore volume of approximately 0.112 cm^3^/g, and a pore size of approximately 39.319 Å. The BET surface for the H_2_O_2_ cement is around 59.11 m^2^/g, the pore volume is approximately 0.080 cm^3^/g, and the pore size ranges between 54 and 65 Å.

**Figure 3:**
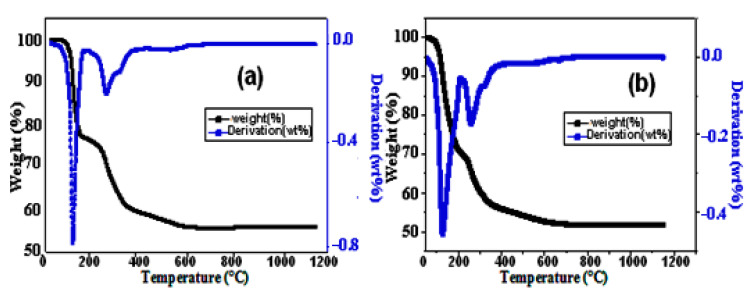
TGA curve of pure (**a**) and H_2_O_2_ (**b**) cements.

Figure 3 depicts the thermogravimetric analyses’ results, which reveal a loss of mass between 100 and 700 °C. Due to the dehydration and decomposition of struvite, the total mass loss for pure cement is 44.26% (a), and for H_2_O_2_ cement it is approximately 48.13% (b). However, the dehydration phenomena identified between 100 and 300 °C for struvite can be expressed as follows:

MgNH_4_PO_4_.6H_2_O (struvite) → MgNH_4_PO_4_ + 6H_2_O (2)

Additionally, from 300 to 700 °C, the decomposition reaction of the material is expressed in terms of

MgNH_4_ PO_4_→ MgHPO_4_ + NH_3_ (g) (3)

**Figure 4:**
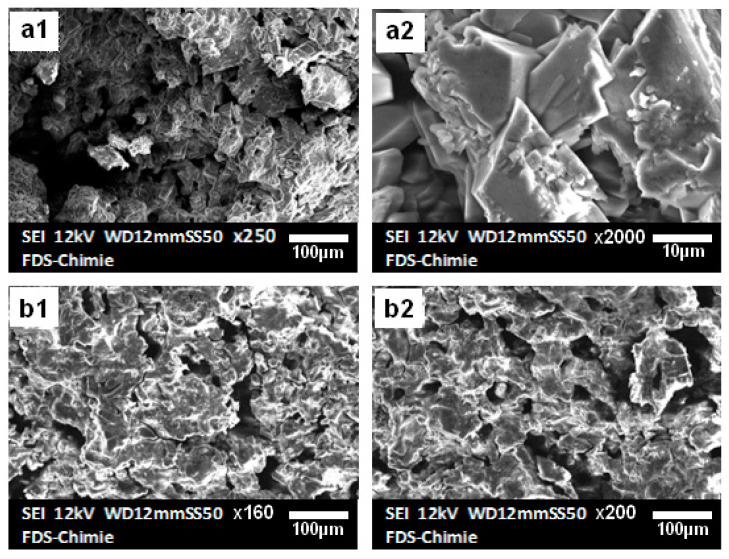
SEM images of pure (**a1**,**a2**) and H_2_O_2_ (**b1**,**b2**) cements.

Figure 4 shows the SEM pictures of the two materials and shows that low porosity struvite crystals have formed. As a result of the rods’ overlap, which creates a small amount of porosity, the sample’s microstructure appears compact and contains tubular crystals with low porosity.

**Figure 5:**
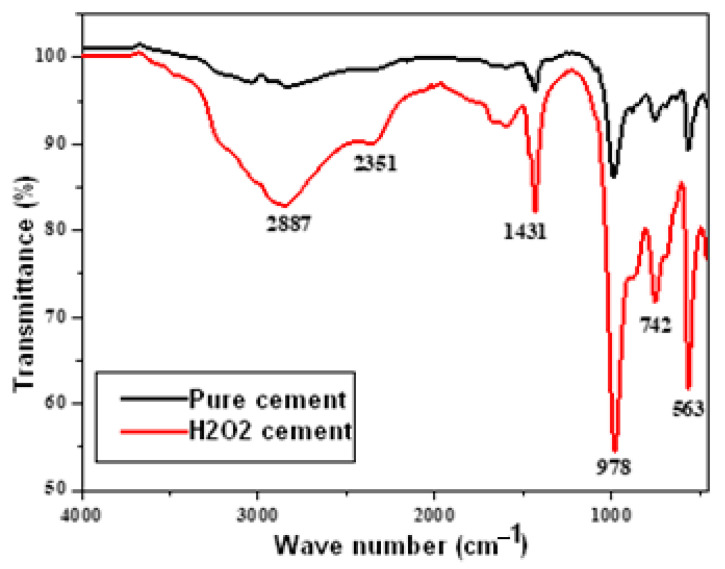
FTIR of pure and H_2_O_2_ cements.

To ascertain the functional groups and the pattern of binding of the various materials, the FTIR was used. Figure 5 shows the two materials’ respective spectra. The H-O-H stretching vibrations of a collection of water molecules engaged in crystallization are responsible for the broad band at 2887 cm^−1^ in the spectrum. The bending modes of the P-O bonds in phosphate groups are responsible for the double peak at 563 and 742 cm^−1^. The P-O vibration asymmetry of ${\mathrm{PO}}_{4}^{3-}$ in the cement was attributable to the adsorption bands at 978 and 2351 cm^−1^. In the vicinity of 1431 cm^−1^, the ${\mathrm{NH}}_{4}^{+}$ group’s distinctive tiny peak could be seen [14,15,16].

### 3.2. New Composites

**Figure 6:**
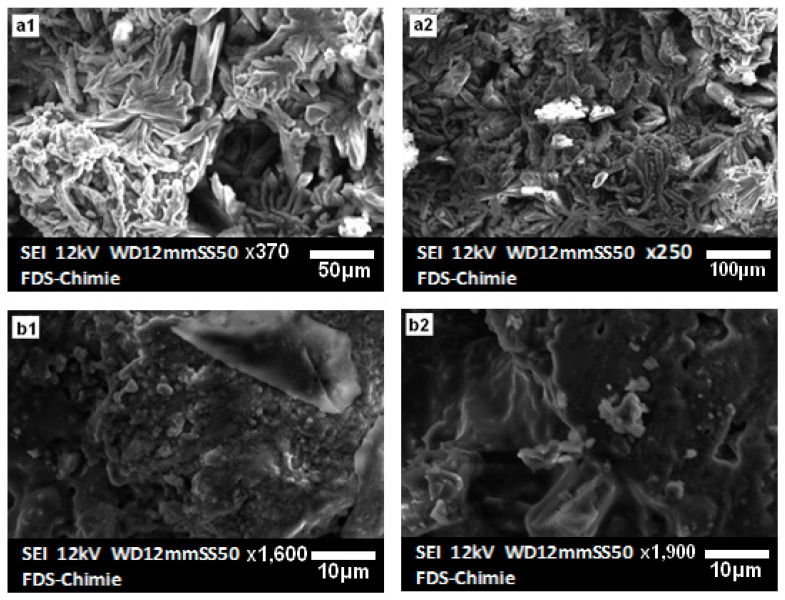
SEM images of zeolite (**a1**,**a2**) and alumina (**b1**,**b2**) cements.

The use of hydrogen peroxide as a solvent had a positive effect on the hardness and strength of the material. The material obtained is harder, and its resistance increases both dry and in water even after a number of days. The stability of the construction was proven by the analyses of the numerous outcomes. This cement, zeolite, and alumina were combined to create new composite materials that could be utilized to retain fluoride. Moreover, due to fluoride’s significant affinity for Al^3+^ and $PO_{4}^{3-}$ ions, these materials may be the best option for removing fluoride from wastewater [17,18]. Figure 6 depicts the SEM images of the composites synthesized with zeolite (Figure 6(a1,a2)) and alumina (Figure 6(b1,b2)). These materials exhibit more interesting properties than pure cement; as the structure becomes harder, the water resistance increases, and the solid remains stable and intact even after more than 15 days of continuous stirring. In addition, the strong gas release observed during synthesis also increases the degree of porosity.

**Figure 7:**
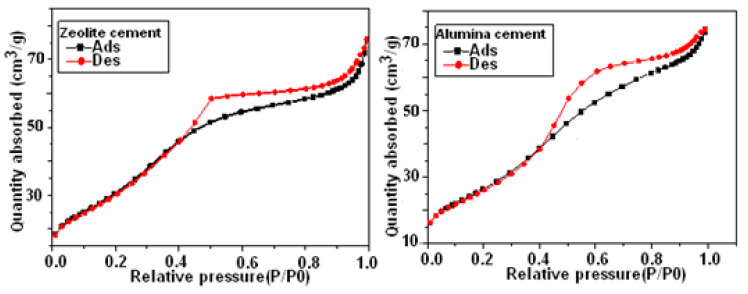
N2-adsorption/desorption curve for zeolite and alumina cements.

Figure 7 illustrates the specific surface that Brunauer, Emmett, and Teller (BET) described. Zeolite cement has a BET surface of around 118.4686 m^2^/g, a pore volume of approximately 0.11753 cm^3^/g, and a pore size of approximately 39.6841 Å. The BET surface for the alumina cement material is around 98.3703 m^2^/g, the pore volume is approximately 0.115104 cm^3^/g, and the pore size ranges from 46.8045 Å. The N2 adsorption/desorption curve for zeolite cement and alumina cement is also shown in Figure 7, and it produces the type II hysteresis curve that is most frequently observed. Multilayer adsorption is related to this kind of isotherm. Since the first layer is entirely saturated at the curve’s inflexion point, multi-layering is possible as the relative pressure rises.

### 3.3. Adsorption

#### 3.3.1. Adsorption Study

**Figure 8:**
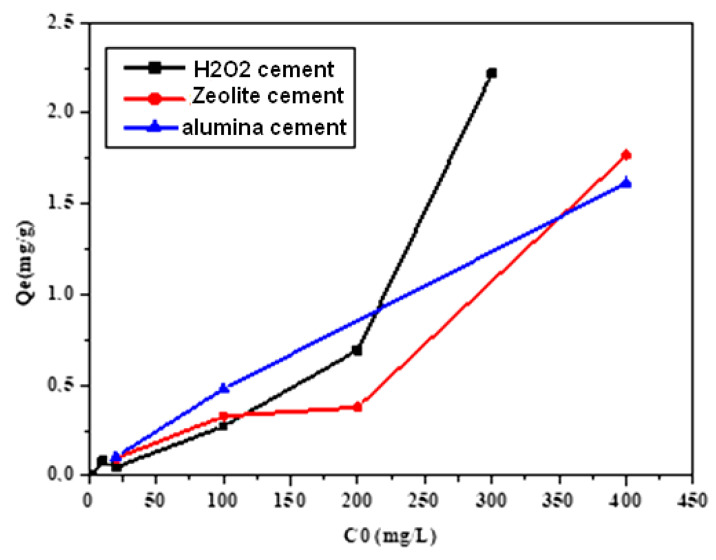
Influence of the initial concentration of fluoride on adsorption.

These solids were tested at various concentrations (1, 5, 10, and 20 mmol/L) to investigate the impact of the three different synthetic materials on the efficacy of fluoride removal. Figure 8 shows the experimental results, which show that fluoride elimination increased as the original concentration was increased. The adsorption of fluoride was carried out at 25.2 °C (room temperature). The fluoride solution in 20 mL tubes was swirled at 200 rpm with a fixed solid mass of 0.3 g for all samples overnight (15 h), and the pH was fixed for all samples. The initial concentrations of the fluoride solution ranged from 2 to 400 mg/L. The filtrate was then examined for residual fluoride content using a particular fluorine electrode after being filtered using 0.2 m filters. Standard fluoride solutions of 2, 10, 20, 100, 200, 300, and 400 mg/L were used for calibrations. The following equation [19,20] is used to compute the quantity of fluoride absorbed, qe (mg/g):

(4) $$q_{e}=\frac{\left(C_{0}-C_{e}\right)\times V\;}{m}$$

where C_0_ and C_e_ are the initial and final concentrations of fluoride in solution (mg/L), V is the volume of solution (L), and m is the mass of adsorbent (g).

As seen in Figure 8, the adsorption capacity increases as the initial concentration increases. The zeolite adsorption curve has a plateau section because the adsorbent and adsorbate have a low affinity for one another. When the initial concentration of zeolite is between 100 and 200 mg/L, the adsorption process slows down. As a result, the amount of adsorbed fluoride slightly increases at these concentration levels. Similar occurrences are shown in another paper [21]. The high diffusion resistance of the pores may make adsorption sites on their inner surface less accessible. In addition, Figure 8 demonstrates that the materials created with H_2_O_2_ alone have the best adsorption values (q_max_ = 2.21 mg/g). Additionally, the adsorbed levels of the compound with zeolite content were higher than those made with alumina: 1.76 mg/g and 1.61 mg/g, respectively.

#### 3.3.2. Equilibrium Studies

The adsorption isotherms show how the degree of adsorption varies with the concentration of the solute. The isotherm gives a clear understanding of how effective various materials are at removing fluoride ions from water. It also shows the maximum amount of fluoride that has been absorbed. Langmuir and Freundlich isotherms were used for equilibrium investigations [19]. A specific kind of monolayer adsorption is indicated by the Langmuir isothermal model. The most typical linearized version is

(5) $$\frac{1}{q_{e}}=\frac{1}{q_{m}}+\frac{1}{bq_{m}C_{e}}$$

where $q_{m}$ is the maximum adsorption capacity (mg/g) and *b* is the Langmuir adsorption constant (L/mg). Plots of $\frac{1}{q_{e}}=f\left(\frac{1}{C_{e}}\right)$ result in a straight line with a slope $\frac{1}{bq_{m}}$ and an intercept $\frac{1}{q_{m}}$.

A form of multilayer adsorption is predicted by the Freundlich isotherm, which also provides a deeper understanding of the adsorbent’s effectiveness and the maximum quantity of adsorbate it can hold. The most used linearized form is

(6) $$\log q_{e}=\log K_{f}+\frac{1}{n}\log C_{e}$$

where *K_f_* (mg/g) is a constant, which approximately indicates the adsorption capacity, and *n* is the function of the strength of adsorption or heterogeneity factor. A plot of $\log q_{e}\;\mathrm{vs}\;\log C_{e}$ results in a straight line with a slope $\frac{1}{n}$ and an intercept$\;\log K_{f}$. The values of *K_f_* and *n* are characteristic of a given adsorbate–adsorbent system. [19,20].

A factor (*A*) in the Temkin isotherm assesses the influence of adsorbate–adsorbent interactions on adsorption. According to the following formula, this shows that the adsorbent heat of all molecules in the layer reduces linearly with increasing adsorbent surface coverage:

(7) $$q_{e}=B\;\ln(a\;C_{e})$$

Linearized in the form

(8) $$q_{e}=\frac{RT}{b}\ln A+\frac{RT}{b}\ln C_{e}$$

where *b* is the Temkin constant linked to the heat of sorption (j/mol), *A* is the Temkin’s isotherm constant (L/mg), *R* is the ideal gas constant (8.314 J/mol K), *T* is the temperature (K), and *C_e_* is the equilibrium concentration (mg/L).

On the other hand, the Van der Waals forces and multilayer nature of adsorption are handled by the Dubinin–Radushkevich isotherm. It is now used to describe how Gaussian energy induces adsorption on heterogeneous surfaces. For various ions present in physical and chemical adsorption, this model can be used. The formula is given below:

(9) $$\ln q\varepsilon =\ln q_{m}-\beta \;\varepsilon^{2}$$

where ε is the Polanyi potential, $\varepsilon =RTln\left(1+\frac{1}{C_{e}}\right)$, and *β* is the adsorption energy. The applicability of different isothermal equations was checked through assessing the R^2^ correlation coefficients [19].

The isotherms of the different composites are outlined in Table 2. With reference to the Table 2, we may infer that the sample H_2_O_2_ cement and zeolite cement adhere to the Langmuir model because their correlation coefficients are R^2^ = 0.973 and 0.974, respectively, while the Freundlich isotherm, with coefficient correlations of R^2^ = 0.999, is the best acceptable model for alumina cement.

#### 3.3.3. Comparison with Other Used Adsorbents

Table 3 compares the various synthetic materials and additional adsorbents created by various researchers in terms of their capacity to remove fluoride. The composite cement outperformed a number of previously reported adsorbents as a defluorinating agent.

## 4. Conclusions

Different materials have been created in the recent study to increase cement resistance in water and use it for fluorine adsorption. According to the study’s experimental findings, H_2_O_2_ appears to be a promising solvent due to both its higher resistance and capacity to remove fluoride from drinking water. New composite cement based on zeolite and alumina was synthesized in order to improve the resistance of cement in water and to use it in the fluoride retention. According to the investigation, the materials created solely with H_2_O_2_ (q_max_ = 2.21 mg/g) have the best adsorption values for the various adsorption materials. Based on the isotherms below as well as the values of the parameters found, and comparing the values of the correlation coefficients R^2^, it can be concluded that the synthesized materials follow the Langmuir model, which is the most appropriate with correlation coefficients R^2^ closer to 1. Moreover, this isotherm is relative to monolayer adsorption and accords with the value of the separation factor R_L_ found, which confirms that the adsorption is favorable. In addition, this cement demonstrates itself to be an intriguing and important molecule that merits further and deeper study because of its beneficial qualities and many applications.

## Figures and Tables

**Table 1:** Chemical compositions of synthesized samples.

	MgO (g)	NH_4_H_2_PO_4_ (g)	Borax (g)	H_2_O (mL)	H_2_O_2_ (mL)	Al_2_O_3_ (g)	Zeolite (g)
Pure cement	1.0780	2.8720	0.3190	2	-	-	-
H_2_O_2_ cement	1.0560	2.8225	0.3270		2	-	-
Zeolite cement	1.600	2.8240	0.3530		2	-	0.54
Alumina cement	1.300	2.8710	0.3110		2	0.54	-

**Table 2:** Values of different parameters of Langmuir, Freundlich, Temkin, and Dubinin–Radushkevich isotherm.

	Equation	H_2_O_2_ Cement	Zeolite Cement	Alumina Cement
Langmuir	$\frac{1}{\;q_{e}}=\frac{1}{q_{m}}+\frac{1}{q_{m}bc_{e}}$	b = −4.322 L/g q^0^ = 0.231 mg/g R_l_ = 0.978 K_ap_ = 0.118 R^2^ = 0.9736	b = 0.9953 L/g q^0^ = 8.340 mg/g R_l_ = 0.064 K_ap_ = 7.22 R^2^ = 0.9743	b = −1.53 L/g q^0^ = 8.34 mg/g R_l_ = 0.064 K_ap_ = 7.22 R^2^ = 0.850
Freundlich	$\log q_{e}=\log K_{f}+\frac{1}{n}\log C_{e}$	1/n = 5.712 K_f_ = 2.926 R^2^ = 0.9426	1/n = −4.99 K_f_ = 2.80 R^2^ = 0.881	1/n = −4.88 K_f_ = 2.80 R^2^ = 0.999
Temkin	$q_{e}=\frac{RT}{b}\ln A+\frac{RT}{b}\ln C_{e}$	A = 0.160 B = 0.373 R^2^ = 0.608	A = 0.160 B = 0.373 R^2^ = 0.160	A = 0.160 B = 0.373 R^2^ = 0.238
Dubinin–Radushkevich	$\ln q\;\varepsilon =\ln q_{m}-\beta \;\varepsilon^{2}$	q^d^ = 0.418 B = −4.108 R^2^ = 0.590	q^d^ = 0.437 B = 1.53 R^2^ = 0.614	q^d^ = 0.43 B = 1.53 R^2^ = 0.849

**Table 3:** Comparative fluoride removal capacity of some adsorbents.

Adsorbent	Reference	Removal Capacity (mg/g)
Lignite	Pekar and al. (2009) [22]	0.71
Modified immobilized activated alumina	Rafique and al. (2012) [23]	0.76
Manganese dioxide-coated activated alumina	Tripathy and Raichur (2008) [24]	1.22
H_2_O_2_ cement	Present study	2.21
Zeolite cement	Present study	1.76
Alumina cement	Present study	1.61

## Data Availability

Data will be requested to the authors.
